# Low-level environmental lead exposure in childhood and adult intellectual function: a follow-up study

**DOI:** 10.1186/1476-069X-10-24

**Published:** 2011-03-30

**Authors:** Maitreyi Mazumdar, David C Bellinger, Matthew Gregas, Kathleen Abanilla, Janine Bacic, Herbert L Needleman

**Affiliations:** 1 Department of Neurology, Children's Hospital Boston, 300 Longwood Avenue, Boston, MA USA; 2 Department of Environmental Health, Harvard School of Public Health, 665 Huntington Avenue, Boston, MA USA; 3 Clinical Research Program, Children's Hospital Boston, 300 Longwood Avenue, Boston, MA USA; 4 University of Pittsburgh School of Medicine, Pittsburgh, PA USA

## Abstract

**Background:**

Early life lead exposure might be a risk factor for neurocognitive impairment in adulthood.

**Objectives:**

We sought to assess the relationship between early life environmental lead exposure and intellectual function in adulthood. We also attempted to identify which time period blood-lead concentrations are most predictive of adult outcome.

**Methods:**

We recruited adults in the Boston area who had participated as newborns and young children in a prospective cohort study that examined the relationship between lead exposure and childhood intellectual function. IQ was measured using the Wechsler Abbreviated Scale of Intelligence (WASI). The association between lead concentrations and IQ scores was examined using linear regression.

**Results:**

Forty-three adults participated in neuropsychological testing. Childhood blood-lead concentration (mean of the blood-lead concentrations at ages 4 and 10 years) had the strongest relationship with Full-Scale IQ (β = -1.89 ± 0.70, p = 0.01). Full-scale IQ was also significantly related to blood-lead concentration at age 6 months (β = -1.66 ± 0.75, p = 0.03), 4 years (β = -0.90 ± 0.41, p = 0.03) and 10 years (β = -1.95 ± 0.80, p = 0.02). Adjusting for maternal IQ altered the significance of the regression coefficient.

**Conclusions:**

Our study suggests that lead exposure in childhood predicts intellectual functioning in young adulthood. Our results also suggest that school-age lead exposure may represent a period of increased susceptibility. Given the small sample size, however, the potentially confounding effects of maternal IQ cannot be excluded and should be evaluated in a larger study.

## Background

Lead is a potent neurotoxicant with demonstrated effects on the brains of children and adults. The weight of evidence supports an association between early life exposure to lead and impaired cognitive function in children[1-3]. Childhood lead exposure might also be a risk factor for neurocognitive impairment in adulthood. Studies have suggested a link between preschool blood-lead concentrations and adult mental retardation [4,5], economic productivity [6,7], delinquency, and violent offences[8,9].

The Boston prospective study was one of several cross-sectional and cohort studies that were initiated in the late 1970 s and early 1980 s to study the relationship between lead and child development. These studies contributed to the decision by the United States Centers for Disease Control and Prevention to announce a new action level of 10 μg/dL whole blood lead in 1991[10]. Follow-up of this cohort at age 10 years showed a continued association between a child's blood-lead concentration at age two years and cognitive function[11]. This finding was influential in experts concluding that children were especially vulnerable to peak blood-lead concentrations, which typically occur at about two years of age.

The objective of this study was to assess the relationship between early life environmental lead exposure and intellectual function in adulthood among a group of young adults who were enrolled in a prospective cohort study as newborns. In addition, we attempted to identify which time period blood-lead concentrations are most predictive of late outcome.

## Methods

### Sample

Between August 1979 and April 1981, a cohort of 249 infants was established among babies born at the Brigham and Women's Hospital in Boston. Umbilical cord blood-lead concentrations were measured, and postnatal blood-lead concentrations and development were assessed at 6, 12, 18, 24, and 57 months, and again at 10 years[11-13]. Follow-up of this cohort at age 10 years consisted of 148 children (87.6% of those considered eligible; 59.4% of the original cohort)[13].

In January 2009, subjects were mailed an introductory letter explaining a new study regarding early-life lead exposure and health outcomes in adulthood. Names and last known address were available only for the 148 subjects who participated in the 10-year follow-up. Of these, 89(60%) were located throughout the United States, and 55 of these (62%) enrolled in the study, which included completing a questionnaire and donating a blood sample. Forty-three subjects came to Children's Hospital Boston (CHB) for additional neuropsychological testing and constitute the sample for the current study. The sample generally consisted of white, college-educated children with college-educated parents (Table 1). Participants were similar to members of the original cohort who did not participate or undergo neuropsychological assessment in terms of demographic factors, measures of socioeconomic status, blood lead history, and IQ scores in early childhood (Table 1).

**Table 1:** Characteristics of the participants at age 28-30 years and comparison with non-participants

Characteristic	Subjects who participated in IQ testing (n = 43)	Subjects who did not participate (n = 105)	p-value
**Subjects**			

Age at testing (years)	29.0 ± 0.5	-	

College graduate^a^	81.4	-	

Currently smoke^a^	23.3	-	

Alcohol use more than 2 drinks/week^a^	34.9	-	

Concussion or head trauma^a^	23.8	-	

Ever arrested^a^	18.6	-	

			

Male^a^	48.8	51.4	0.77

White^a^	93.0	91.2	0.70

First born^a^	53.5	58.7	0.59

Weeks of gestation	40.0 ± 1.7	40.0 ± 2.1	0.96

Birth weight (kg)	3.4 ± 0.5	3.4 ± 0.5	0.76

			

Blood lead concentration (μg/dL)^b^			

Cord	6.5 ± 5.3	7.3 ± 5.3	0.43

6 months	8.0 ± 5.3	8.4 ± 7.5	0.74

12 months	10.0 ± 6.7	9.6 ± 6.5	0.72

24 months	7.7 ± 4.0	8.9 ± 6.9	0.28

4 years	6.7 ± 3.6	6.3 ± 4.3	0.64

10 years	3.0 ± 2.7	2.9 ± 2.3	0.79

IQ at age 4 years^b^	117.7 ± 15.3	114.6 ± 14.3	0.22

IQ at age 10 years^b^	117.7 ± 15.2	115.3 ± 13.9	0.36

			

**Subjects' mothers**			

Age at delivery (year)^b^	30.3 ± 4.3	30.1 ± 4.7	0.82

College graduate^a^	60.0	59.3	0.49

Maternal IQ^b^	122.8 ± 19.3	122.4 ± 17.8	0.90

HOME score^b^	51.4 ± 4.4	51.0 ± 3.9	0.60

Tobacco use during pregnancy^a^	27.5	26.9	0.94

Alcohol use during pregnancy^a^	55.0	42.1	0.15

^a^(% Yes) ^b^Mean ± SD

### Neuropsychological Assessment

A child neurologist (M.M.), trained in the administration of the instrument and unaware of the subject's developmental history and lead concentrations, administered the Wechsler Abbreviated Scale of Intelligence (WASI) to all subjects. The WASI includes four subtests (Vocabulary, Similarities, Block Design and Matrix Reasoning) and provides estimates of Full-Scale IQ, Verbal IQ, and Performance IQ[14]. A senior neuropsychologist (D.C.B.) reviewed 25% of the WASI protocols to ensure accurate administration and scoring.

### Measurement of Potential Confounders

At the time of enrollment in the current study, subjects completed a questionnaire that gathered demographic information as well as information about education, employment, history of arrests, concurrent medications, and alcohol and tobacco use. During previous assessments, parents completed several questionnaires about the child's medical history and sociodemographic characteristics, including the Home Observation for Measurement of the Environment (HOME) Inventory[15]. The HOME score is an index reflecting the quality and quantity of emotional and cognitive stimulation in the child's environment. Information about many other potential confounders was available in records from earlier assessments (e.g., maternal IQ, birth weight).

### Measures of exposure

Blood samples were obtained from umbilical cords at birth and directly from subjects using capillary tubes at ages 6, 12, 18, and 24 months and via venipuncture at 57 months and 10 years. Blood lead concentrations were measured in duplicate using graphic furnace atomic absorption spectrometry with quality control samples included among sample batches. The details of this method are available in previous reports from this cohort[12,13].

### Covariates

All analyses included prespecified covariates, which consisted of established predictors of children's intellectual outcomes, factors widely used in studies of lead exposure and intelligence, as well as factors in early adulthood that have been shown to affect intellectual outcome. The following variables were used: subject's sex, race, birth weight, birth order, gestational age at delivery, mother's marital status at delivery, highest educational level of mother at delivery, maternal IQ, maternal tobacco use during pregnancy, maternal alcohol use during pregnancy, subject's history of concussion or head trauma, subject's smoking, and subject's use of alcohol.

### Statistical Methods

As predictors of IQ in adulthood, we considered several blood-lead concentrations: umbilical cord blood lead, six months, one year, two years, four years and 10 years, average blood-lead concentration (defined as the mean blood-lead concentration between birth and 10 years), maximum blood-lead concentration (defined as the highest blood-lead concentration measured from birth to 10 years), average late childhood blood-lead concentration (defined as the mean of four year and 10 year blood-lead concentrations), and early childhood blood-lead concentration (defined as the mean blood-lead concentration from six months to two years and as the mean blood-lead concentration from birth to two years). Spearman correlations were computed for lead and covariates and for IQ and covariates. We fit separate linear regression models with each lead measure as the predictor and IQ as the response. We further attempted to determine the pattern of exposure's influence on IQ by specifying a model that contained terms for the ratio of 10 year (and subsequently, four year) blood-lead concentration to the two year blood-lead concentration in addition to the average late childhood blood concentration.

The lead-IQ models describe only the marginal relationship between blood-lead concentrations and IQ and do not control for the effect of confounders. The small sample size prevented us from fitting a multiple regression model with all the variables plus lead concentrations as predictors. We did, however, want to determine which of these covariates affected the relationship between blood-lead concentrations and IQ. Thus we added each covariate one at a time to the model with lead as the sole predictor. If the significance of the regression coefficient for the blood lead measure changed from below 0.05 to above 0.05 as a result of adding the candidate covariate to the model, we labeled the term to be a possible confounder that would have to be accounted for in a larger study. All model assumptions were validated with residual plots.

## Results

### Blood lead concentration

**Figure 1:**
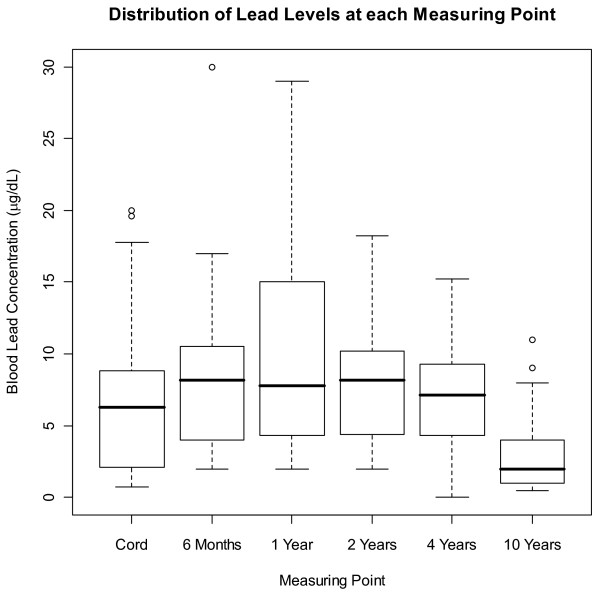
**Distribution of lead levels at each measuring point**. In each box plot, the median value is indicated by the center horizontal line and the 25^th ^and 75^th ^percentiles are indicated by the lower and upper horizontal lines, respectively. The vertical lines represent 1.5 times the interquartile range, and circles represent outliers (i.e. values that are more than 1.5 times the interquartile range). To convert values for lead to micromoles per liter, multiply by 0.0483.

The mean blood lead concentration was lowest at the age of 10 years (3.0 μg/dL), and was maximal at age two years (10.0 μg/dL) (Table 1). The median concentrations followed a similar pattern (Figure 1). The highest concentrations were seen in infancy and early childhood, possibly reflecting greater lead intake through hand-to-mouth activity and higher exposures in that era. Only five subjects had blood-lead concentrations at each measuring point that were below 10.0 μg/dL, and so separate analyses for subjects whose lead exposures were below 10.0 μg/dL were not possible. In general, however, subjects in our study had lower lead exposures than similar cohorts of the time period

### Intelligence test results

The mean Full-Scale IQ in our sample was approximately 122 at age 29 years. Adult IQs were strongly correlated with IQ scores at the ages of 57 months and 10 years (r = 0.68, p < 0.001 and r = 0.67, p < 0.001 respectively). IQ scores in early adulthood were also strongly correlated with maternal IQ (r = 0.63, p < 0.001). The bivariate associations between the subject's IQ, blood lead concentration and other covariates were in the expected direction.

### Blood lead concentrations and IQ

We examined the relationship between Full-Scale IQ and eleven blood lead measures. Full-Scale IQ was significantly related to blood-lead concentration at 6 months (p = 0.03), four years (p = 0.04), 10 years (p = 0.020), average blood-lead concentration (p = 0.03), and average late childhood blood-lead concentration (p = 0.01). Although all these measures are related to Full-Scale IQ, average late childhood blood lead (defined as the mean of blood-lead concentrations at age four years and age 10 years) had the strongest relationship with Full-Scale IQ as measured by regression coefficient and *R^2^*. Average late childhood blood lead and average blood lead were used in all subsequent analyses as the primary lead exposure indices because both of these measures were related to Full-Scale IQ and also took into account the lead concentrations at each age that were also shown to be related to Full-Scale IQ.

Similar findings were seen in analyses of Performance and Verbal IQ (Table 2). Performance IQ was significantly related to blood-lead concentration at 6 months (p = 0.03), 4 years (p = 0.02) and average blood-lead concentration (p = 0.02). Verbal IQ was significantly related to blood-lead concentration at 10 years (p = 0.001). As with Full-Scale IQ, the blood-lead concentrations in childhood had stronger associations with Performance and Verbal IQ than blood-lead concentrations at earlier ages. For subsequent analyses, we decided to focus on Full-Scale IQ to facilitate comparison with other studies.

**Table 2:** Unadjusted Changes in IQ for Each Increase in the Blood Lead Concentration of 1 microgram per Deciliter

Type of Blood Lead		Full-Scale IQ			Verbal IQ			Performance IQ		
Measurement	*n*	*β ± SE*	*p*	R^2^	*β ± SE*	*p*	R^2^	*β ± SE*	*p*	R^2^
**Average late childhood**	42	-1.89 ± 0.70	0.01	0.154	-1.98 ± 0.76	0.12	0.145	-1.33 ± 0.66	0.05	0.091
(mean of 4 yr and 10 yr)		(-3.30, -0.47)			(-2.68, 0.01)			(-2.68, 0.01)		

**Average**	43	-1.66 ± 0.75	0.03	0.106	-1.22 ± 0.83	0.15	0.050	-1.68 ± 0.68	0.02	0.129
(mean of all available)		(-3.18, -0.14)			(-3.06, -0.30)			(-3.06, -0.30)		

**6 months**	39	-0.90 ± 0.41	0.03	0.117	-0.80 ± 0.45	0.08	0.080	-0.81 ± 0.37	0.03	0.115
		(-1.73, -0.77)			(-1.71, 0.11)			(-1.57, -0.06)		

**4 years**	40	-1.26 ± 0.58	0.04	0.110	-0.96 ± 0.64	0.14	0.055	-1.25 ± 0.51	0.02	0.136
		(-2.43, -0.09)			(-2.27, 0.35)			(-2.29, 0.22)		

**10 years**	37	-1.95 ± 0.80	0.02	0.145	-2.84 ± 0.81	0.001	0.258	-0.52 ± 0.72	0.48	0.520
		(-3.56, -0.33)			(-4.49, 1.19)			(-1.98, 0.99)		

The relationship between blood lead indices and Full-Scale IQ was determined to be linear. Previous literature suggests that the logarithm of lead concentration is a better linear predictor of IQ than lead on the natural scale[3,16,17]. In our data set we found that taking the logarithm did not improve the fit so we chose to express lead on its measured scale. Furthermore, the model with logarithm of lead did not improve model fit as determined by the Akaike Information Criterion (AIC) and the model residuals.

We attempted to test whether the pattern of exposure is an important modifier of lead exposure following the methods developed by Hornung et al[18]. We added the ratio of 10 year (and subsequently four year) blood-lead concentration over two year blood-lead concentration to the model with average late childhood blood-lead concentration. The parameters associated with the ratio terms were not significant and therefore were not included in further testing. There are several variables that may affect the relationship between Full-Scale IQ and blood-lead concentrations. For example, inclusion of maternal IQ in the model affected the relationship between Full-Scale IQ and average childhood blood-lead concentration (Table 3). When maternal IQ was added to the model the coefficient for the childhood blood lead index was reduced from -1.89 (-3.30, -0.47, 95% CI) to -1.11 (-2.29, 0.06, 95% CI) and the significance of the regression coefficient changed.

**Table 3:** Unadjusted model and models adjusted for important covariates

Covariate	N (Undaj. Model)	N (Adj. Model)	Adjusted estimate for lead [β (95% CI)]	Spearman Correlation between lead level and covariate	Spearman Correlation between Total IQ and covariate
None	42	-	-1.89 (-3.30, -0.47)	-	-

Gender	42	42	-1.88 (-3.31, -0.45)	0.02	0.01

Birth Weight	42	42	-1.46 (-2.69, -0.24)	-0.13	0.56

Birth Order (first born/not first born)	42	42	-1.74 (-3.18, -0.30)	0.17	-0.21

Gestational age	42	42	-1.88 (-3.25, -0.52)	0.03	0.30

Mother's marital status (married/not married)	42	39	-2.00 (-3.57, -0.43)	0.20	-0.28

Mother's education (college/no college)	42	39	-1.89 (-3.33, -0.45)	-0.25	0.39

**Maternal IQ**	**42**	**42**	**-1.11 (-2.29, 0.06)**	**-0.24**	**0.67**

Race (white/nonwhite)	42	42	-1.76 (-3.35, -0.17)	-0.37	0.29

Maternal Smoking During pregnancy (y/n)	42	39	-1.62 (-3.04, -0.21)	0.29	-0.51

Maternal Alcohol Use During pregnancy (y/n)	42	39	-2.28 (-3.88, -0.68)	0.09	-0.03

HOME score (mean of all collected)	42	42	-1.45 (-2.89, -0.01)	-0.25	0.38

Concussion (y/n)	42	41	-1.84 (-3.32, -0.36)	-0.19	-0.08

Subject current smoking (y/n)	42	42	-1.56 (-2.97, -0.14)	0.24	-0.38

## Discussion

Three findings from this study support the hypothesis that there are long-term consequences of environmental lead exposure among children in the United States. Of primary importance is that intellectual functioning in adulthood, as measured by Full-Scale IQ, is inversely associated with blood lead concentrations in childhood. This suggests that the adverse effects of early-life lead exposure are persistent. We recruited adults aged 28 to 30 years who, as newborns, participated in a prospective study of lead and neurodevelopment. This length of follow-up is well past the time horizon of most epidemiological studies. The findings of this study are consistent with other investigations that have found that early exposure to lead is associated with long-term and apparently irreversible effects on behavioral, cognitive, and neuroradiological endpoints in adults[8,19-23].

The second, related finding is that the association between IQ and blood-lead concentration was seen in a cohort with generally lower body lead burden than other cohorts assembled during that era, and lower blood lead concentrations than the current level of concern set by the CDC and WHO[24,25]. This finding is consistent with several other cohort studies that examined the relationship of blood lead concentrations and cognitive development (IQ or psychometric intelligence) in children. For example, in an analysis of the Rochester Longitudinal study [17], the change in IQ per given change in lead concentration was greater among children whose maximal blood lead concentration remained below 10 μg/dL than it was among those children with a maximal blood lead concentration greater than 10 μg/dL. In a subsequent follow-up assessment of this cohort, children with mean blood lead concentrations between 5.0 and 9.9 μg/dL scored 4.9 IQ points lower than children with blood-lead concentrations below 5.0 μg/dL[26].

The third notable finding from our study is that lead concentrations during the school age years are related to IQ in adulthood, and may represent a time period of greater susceptibility to environmental lead exposure, or more stable blood lead body burden. Previous studies using this same cohort of children showed that children's IQ scores and their scores on other neurocognitive tests were much more strongly associated with blood-lead concentrations at age 24 months than with blood-lead concentrations at other ages [13]. This observation supported the hypothesis that this time period represented a window of special vulnerability to lead. More recent studies, however, suggest that lead exposure at school age may be more strongly related to performance on cognitive testing[27,28]. A pooled analysis of seven cohorts [3] found that concurrent lead concentrations have the strongest relationship to IQ, but that early childhood average, lifetime childhood average and peak blood-lead concentrations also had an inverse relationship to IQ in late childhood. Our study uses both average lifetime lead exposure and childhood lead exposure (average of lead concentrations at age four and 10 years), with the stronger association found in the measure using blood-lead concentrations at older ages.

Our study has a number of important limitations, the most important of which is its small sample size. The small numbers of subjects limited our ability to perform multivariate analysis and evaluate the effect of important potential confounders or interactions. We were able to evaluate a number of potentially confounding variables, including the HOME score, maternal education and maternal IQ, on which information was collected prospectively. Adjusting for maternal IQ altered the significance of the regression coefficient for blood-lead concentration. With this small sample size, however, it is difficult to state that maternal IQ explains all the variability in subjects' IQ, that is, that maternal IQ confounds the effect seen in the unadjusted model. An alternative explanation is that maternal IQ is collinear with lead exposure (mothers with higher IQs took active steps to limit lead exposure, or lived in housing with less lead exposure) and therefore should not be used in models. In any case, our finding that average late childhood blood-lead concentrations are associated with IQ in adulthood should be replicated in other cohorts with larger sample sizes.

## Conclusions

Our study suggests that lead exposure in childhood predicts intellectual functioning in young adulthood. The results also suggest that school-age lead exposure may represent a period of increased susceptibility. Given the small sample size, however, the potentially confounding effects of maternal IQ cannot be excluded and should be evaluated in a larger study.

## Abbreviations

HOME: Home Observation for Measurement of the Environment; IQ: intelligence quotient; WASI: Wechsler Abbreviated Scale of Intelligence.

## Competing interests

The authors declare that they have no competing interests.

## Authors' contributions

The study was designed by MM, DCB and HLN. MM and DCB performed the IQ assessments. KA, MM, MG and JB performed the statistical analysis. The first draft of the manuscript was written by MM and comments and changes were made by KA, MG, JB, and DCB. All authors have approved the final manuscript.
